# Supplementary figures and images for: Ring canals in the larval adipose of Drosophila buffer stress response
Source: bioRxiv. 2025 Jun 11:2025.06.11.658881. Preprint. [Version 1] doi: 10.1101/2025.06.11.658881 (PMC12259129; doi:10.1101/2025.06.11.658881)

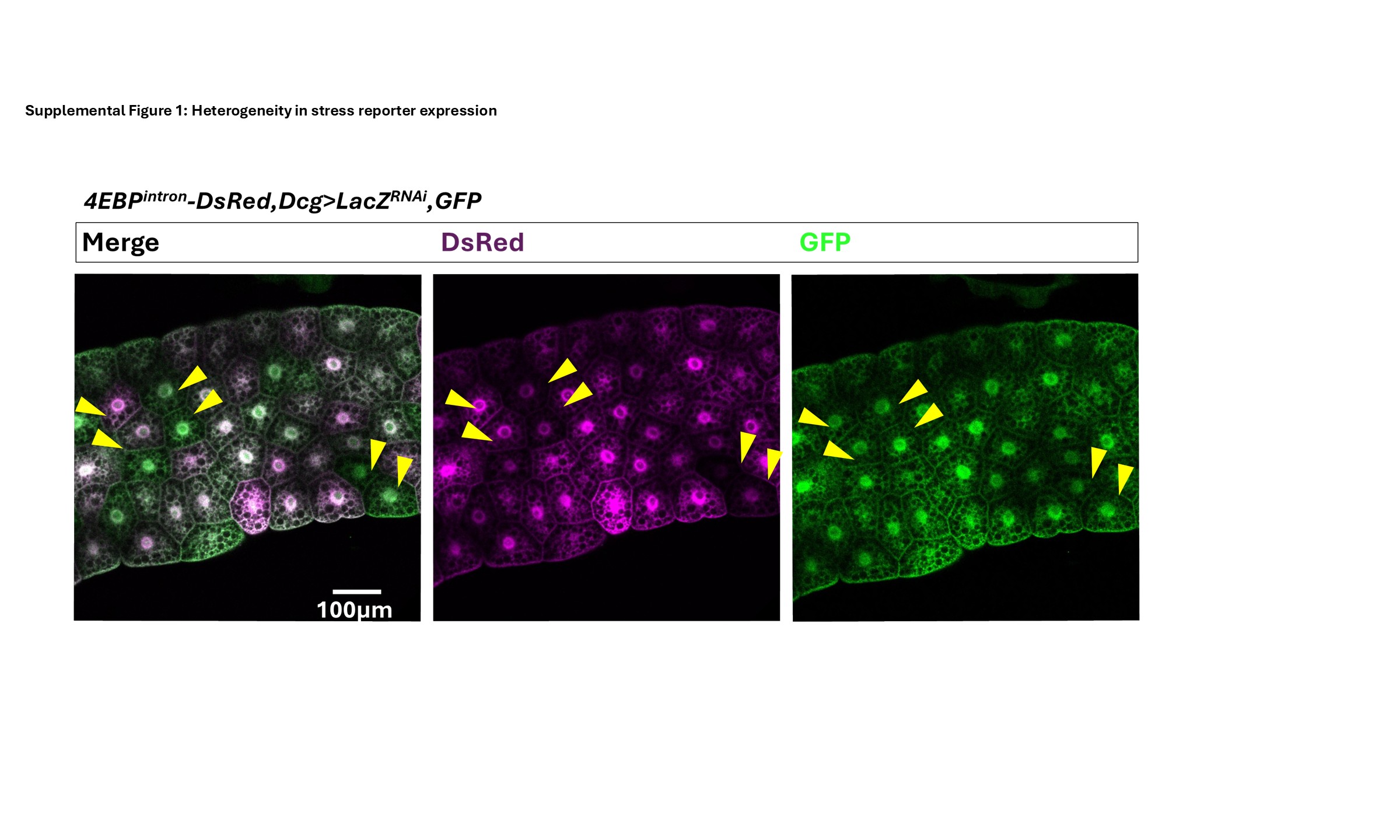

Supplement: Supplement 1 [file media-1.jpg]
